# Supplementary figures and images for: Characterizing the social media footprint of general surgery residency programs
Source: PLoS One. 2021 Jun 30;16(6):e0253787. doi: 10.1371/journal.pone.0253787 (PMC8244871; doi:10.1371/journal.pone.0253787)

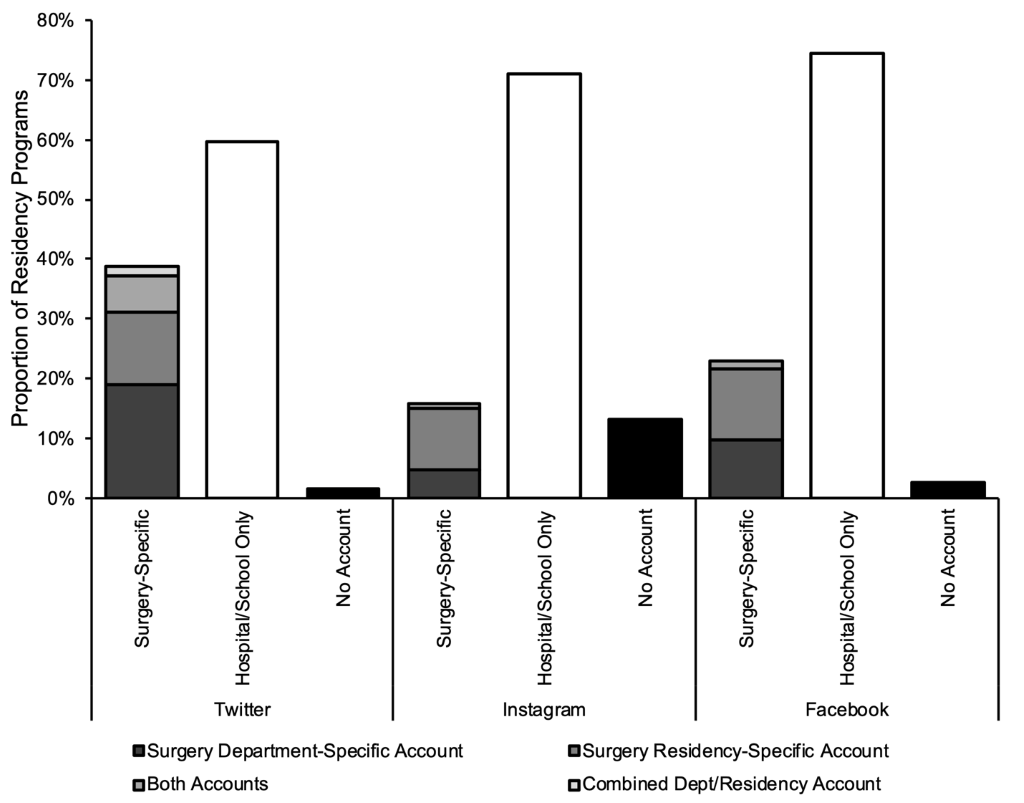

Supplement: S1 Fig — Most general surgery residency programs have an affiliated surgery-specific account and/or hospital, healthcare system, or medical school account. Surgery-specific accounts include Department of Surgery accounts, residency program accounts, and accounts which were identified as combined departmental and residency accounts. Some programs had both separate departmental and residency accounts. (TIF) [file pone.0253787.s001.tif]
