# Supplementary material for: Characterizing the social media footprint of general surgery residency programs
Source: PLoS One. 2021 Jun 30;16(6):e0253787. doi: 10.1371/journal.pone.0253787 (PMC8244871; doi:10.1371/journal.pone.0253787)
Supplement: S1 Table — Recent social media activity, as measured by median (interquartile range [IQR]) days since most recent post, for independent, university-affiliated, and university-based U.S. general surgery residency programs. Row p-values (Kruskal-Wallis tests) are noted. (DOCX) [file pone.0253787.s002.docx]

**S1 Table.** **Recent social media activity by account type and program type.**

| **Account type** | **Days since last post on account, median (IQR)** | | | **p-value (Kruskal-Wallis)** |
| --- | --- | --- | --- | --- |
|  | **Independent**  **(n=63)** | **University-affiliated**  **(n=84)** | **University-based**  **(n=171)** |  |
| **Twitter** | | | | |
| Hospital and/or medical school | 1 (0-3) | 0 (0-2) | 1 (0-3) | 0.555 |
| Surgery-specific | 14 (2-110) | 26 (9-365) | 4 (1-19) | 0.005 |
| **Instagram** | | | | |
| Hospital and/or medical school | 1 (0-1) | 1 (0-4) | 1 (0-3) | 0.160 |
| Surgery-specific | 4 (4-4) | 32 (7-106) | 11 (4-51) | 0.399 |
| **Facebook** | | | | |
| Hospital and/or medical school | 1 (0-2) | 1 (0-2) | 1 (0-2) | 0.957 |
| Surgery-specific | 31 (2-954) | 2 (1-17) | 23 (4-324) | 0.192 |

Recent social media activity, as measured by median (interquartile range [IQR]) days since most recent post, for independent, university-affiliated, and university-based U.S. general surgery residency programs. Row p-values (Kruskal-Wallis tests) are noted.
