# Supplementary material for: Characterizing the social media footprint of general surgery residency programs
Source: PLoS One. 2021 Jun 30;16(6):e0253787. doi: 10.1371/journal.pone.0253787 (PMC8244871; doi:10.1371/journal.pone.0253787)
Supplement: S2 Table — Social media accounts and activity for general surgery residency program directors (PDs) and assistant or associate program directors (APDs). Up-to-Date LinkedIn profiles included a current position and/or employer as well as a photograph. (DOCX) [file pone.0253787.s003.docx]

**S2 Table. Social media presence of general surgery residency program leadership.**

| **Program leadership** | **Twitter** | | **LinkedIn** | |
| --- | --- | --- | --- | --- |
|  | **Identifiable account** | **Days since last post, median (IQR)** | **Up-to-date account** | **Outdated account** |
| Total (n=609) | 191 (31.4%) |  | 141 (23.2%) | 192 (31.5%) |
| PDs (n=313) | 107 (34.2%) | 14 (4-287) | 82 (26.2%) | 97 (31.0%) |
| APDs (n=296) | 84 (28.4%) | 39.5 (4-443) | 59 (20.0%) | 95 (32.1%) |

Social media accounts and activity for general surgery residency program directors (PDs) and assistant or associate program directors (APDs). Up-to-Date LinkedIn profiles included a current position and/or employer as well as a photograph.
